# Predictors of disease progression in pancreatic neuroendocrine tumors after surgery

**DOI:** 10.1007/s13304-026-02521-0

**Published:** 2026-02-16

**Authors:** Eva Maria Dobrindt, Janina Maren Krömer, Martina T. Mogl, Agata Dukaczewska, Charlotte Friederieke Müller-Debus, Peter Steinhagen, Uli Fehrenbach, Thomas Malinka, Johann Pratschke, Frederike Butz

**Affiliations:** 1 Department of Surgery, Campus Charité Mitte | Campus Virchow Klinikum, Charité—Universitätsmedizin Berlin, Corporate Member of Freie Universität Berlin and Humboldt-Universität zu Berlin, Berlin, Germany; 2 Department of Hepatology and Gastroenterology, Campus Charité Mitte, Campus Virchow Klinikum, Charité—Universitätsmedizin Berlin, Corporate Member of Freie Universität Berlin and Humboldt-Universität zu Berlin, Berlin, Germany; 3 Department of Radiology, Charité—Universitätsmedizin Berlin, Corporate Member of Freie Universität Berlin and Humboldt-Universität zu Berlin, Berlin, Germany

**Keywords:** Pancreatic neuroendocrine tumors, Pancreatic neuroendocrine neoplasms, Oncological outcome, Prognostic factors, Risk stratification

## Abstract

Pancreatic neuroendocrine tumors (pNETs) are a heterogeneous group of tumors that exhibit a range of biological behaviors, from indolent to aggressive. Identifying prognostic factors for pNETs is essential for risk stratification and personalized treatment planning. Clinicopathological data of stage I to III patients who underwent pancreatic resection and were treated for pNET at an ENETS Center of Excellence from 2009 to 2023 were analyzed retrospectively. 89 pNET patients were included (45 female, 44 male). Proximal pNETs had higher proportions of lymph node metastases (48.4% vs. 17.2%, *p* = 0.002) and shorter progression-free survival (PFS) compared to distal tumors (5-year PFS 56.2% vs. 85.0%, *p* = 0.003). Subdivision of G2 into G2a (Ki67 3-<10%) and G2b (Ki67 10–20%) identified shorter PFS in G2b than in G1 and G2a tumors (5-year PFS G1: 93.6%, G2a: 73.5%, G2b: 34.3%, *p* < 0.001) while PFS did not differ between G2b and G3 (34.3% vs. 28.6%, *p* = 0.192). Cox regression analyses revealed tumor grading (G2b: HR 8.396 (2.025–34.081), *p* = 0.003; G3: HR 17.649 (3.912–79.627), *p* < 0.001) and lymph node metastases (HR 2.657 (1.040–6.789), *p* = 0.041) as risk factors for shorter PFS.Tumor grading and lymph node metastases are independent predictors of postoperative disease progression in pNET patients without distant metastases. Subdividing G2 tumors into G2a and G2b improves prognostic discrimination, with G2b tumors demonstrating outcomes comparable to G3 tumors. These findings support the incorporation of refined grading into treatment and follow-up considerations.

**Supplementary Information:**

The online version contains supplementary material available at 10.1007/s13304-026-02521-0.

## Introduction

Pancreatic neuroendocrine tumors (pNETs) are a heterogenous group of rare neoplasms with varying biological behavior. In general, pancreatic neuroendocrine neoplasms are categorized into well-differentiated neoplasms and poorly differentiated neuroendocrine carcinomas (NECs) [1]. Further classification of well-differentiated NETs is based on Ki-67 index and mitotic count into low-grade NET (G1, Ki-67 < 3%), medium-grade NET (G2, Ki-67 3–20%) and high-grade NET (G3, Ki-67 > 20%) [1]. Just like other gastroenteropancreatic (GEP) NETs, the incidence of pNETs has been rising over recent decades, probably due to a higher detection rate of incidental tumors, improved diagnostics and a potential genuine rise in disease incidence [2, 3]. Most pNETs occur as asymptomatic, sporadic tumors while functional pNETs can be associated with inherited syndromes, such as MEN1 and Von-Hippel-Lindau (VHL) syndrome. Though recent evidence has shown that surveillance may offer a therapy alternative for well-selected non-functional pNETs (nf-pNETs) smaller than 2 cm [4–7], surgical resection remains the only curative treatment. As minimally invasive approaches to pancreatic malignancies have become more and more established, pNETs may also be treated by laparoscopic or robotic-assisted surgery in selected cases [8]. To choose the right treatment for the right patient, the identification and knowledge of prognostic factors is crucial. In this context, previous studies have identified different characteristics associated with oncological outcome and tumor aggressiveness in pNETs. For example, tumor size, microvascular invasion, lymph node involvement, tumor grading and Ki-67 index have been associated with poor outcome [9–13]. In addition, the redefinition of cut-off values for Ki-67 index subdividing the large and heterogeneous G2 group and therefore improve the predictive value of tumor grading has also been discussed for pNETs [9, 14].

While current guidelines do not recommend (neo-)adjuvant treatment in resectable pNETs in general, it might be considered in patients at high risk for disease recurrence [15]. In the current study we therefore aimed to identify risk factors for impaired oncological outcome in pNET patients undergoing surgery, focusing on postoperative disease recurrence or progression.

## Methods

Patients diagnosed with pNET who were treated at our institutional European Neuroendocrine Tumor Society (ENETS) Center of Excellence between May 2009 and January 2023 were identified from our Comprehensive Cancer Center database. All patients with well-differentiated pNETs (G1-G3) without distant metastases who underwent primary resection were included. Exclusion criteria for further analyses were primary tumor locations other than the pancreas, stage IV tumors (*n* = 24), patients with functional tumors (*n* = 22), patients receiving enucleations or segmental resections (*n* = 4), patients with hereditary syndromes (*n* = 8) and cases without follow-up data (*n* = 9).

Medical charts were reviewed for demographic and clinical data including age, sex, American Society of Anesthesiologists (ASA) status, Body Mass Index (BMI), distant metastasis status, and tumor-associated symptoms. Pathological reports were reviewed for tumor size, number of resected and positive lymph nodes and resection margin status (R status). Preoperative tumor stage was defined according to the 8th edition of the UICC classification of malignant tumors [16] and tumor grading according to the World Health Organization (WHO) grading system [1]. In addition, a recently proposed alternative grading system [9] was applied, differentiating between the following groups based on Ki-67 index: G1 ≤ 2%, G2a > 2 to < 10%, G2b ≥ 10% to ≤ 20% and G3 > 20%. Information about the surgical approach including open and minimally invasive surgery (MIS) and the surgical procedure were retrieved from surgical reports. Surgical resection techniques included pylorus preserving pancreaticoduodenectomy (PPPD), distal pancreatectomy (DP) and total pancreatectomy. Depending on tumor location, patients were grouped into either proximal *(head*) or distal *(body and tail).* Medical records were also searched for follow-up data including clinical, radiological, or histopathologic evidence of tumor recurrence or progression when applying as well as information about additional therapies including somatostatin analogues (SSA), chemotherapy, everolimus, peptide receptor radionuclide therapy (PRRT), transarterial embolization (TAE), selective internal radiation therapy (SIRT) and brachytherapy.

For further analysis, patients were grouped to those with and without progression. The group with progression includes all individuals, having developed either recurrence or tumor progression.

### Statistical analysis

Continuous variables are summarized as medians with ranges, while categorical variables are reported as frequencies. Comparisons between groups for continuous data were conducted using the Mann-Whitney U test. For categorical data, either the chi-squared test or Fisher’s exact test were applied, as appropriate. Survival analyses were carried out using the Kaplan-Meier method, with survival curves compared via log-rank tests. Progression-free survival (PFS) was defined as the time from pancreatic surgery to the first documented postoperative recurrence or progression. Overall survival (OS) was defined as the time interval from pancreatic resection to death. Patients who had not reached the respective endpoints or were lost to follow-up were censored at their last recorded visit. To compare patients with proximal and distal tumors, a one-to-one propensity score matching using a logistic regression model with a match tolerance of 0.1 was performed based on age and UICC tumor stage. Univariate Cox regression analysis was performed for identification of potential influencing factors of PFS displayed as hazard ratio (HR) with 95%-confidence interval (95%-CI). Factors with significant effect in univariable analysis showing the highest HR and lowest p-value (≤ 0.001) were included in multivariable Cox regression analysis. As only *n* = 25 events (disease progression) were recorded, the number of variables included in the multivariable analysis was limited to 3.

Statistical analyses were performed using SPSS Statistics software, version 29 (IBM Armonk, NY, USA). Figures were created with GraphPad Prism Version 10.0.3 (GraphPad Software, LLC, Boston MA, USA). Missing data were indicated and were excluded analysis by analysis. The significance level was set to 0.05.

The study was approved by the local Institutional Ethics Committee (EA2/064/09) and was conducted in accordance with the Declaration of Helsinki. Patient consent was waived due to the retrospective and non-interventional character of the study.

## Results

Distinct clinicopathological profiles in pNET patients with and without postoperative disease progression.

89 patients were included in the study and their respective characteristics are shown in Table 1. To identify factors associated with disease recurrence and postoperative disease progression, a comparison was performed between patients with and without progression. The respective results are also shown in Table 1.

Patients with or without postoperative disease progression did not show significant differences in sex, age, ASA status or BMI. In patients with progression, the rate of disease-related symptoms was higher (39.0% vs. 18.8%, *p* = 0.025). Additionally, more than 60% of the patients with progression were diagnosed in stage III (*n* = 16, 64.0%) whereas patients without progression mostly presented initially with stage I (*n* = 28, 43.8%) and stage II (*n* = 28, 43.8%). Tumor location differed significantly between patients with and without progression: while 75% of patients without progression had distal tumors, tumors in patients with progression were mostly proximal tumors (*n* = 15, 60.0%). Accordingly, the type of resection differed. Most patients without progression received a DP (*n* = 48, 75.0%), whereas 36.0% of patients presenting with progressive disease received a DP (*p* = 0.001). In general, higher tumor grading was prevalent in patients with progression. More specifically, these patients had significantly more often G2 and G3 tumors (G2: 60.0% vs. 35.9%; G3: 24.0% vs. 1.6%; *p* < 0.001) as well as higher proportions of G2b (28.0% vs. 6.3%), *p* < 0.001, in the alternative grading classification. Other factors that were more predominant in patients with postoperative disease progression included larger tumor size (in mm: 37 (15–140) vs. 18 (2-120), *p* < 0.001) and accordingly higher T stage (T3: 60.0% vs. 21.9%, *p* = 0.002), presence of lymph node metastases (69.0% vs. 12.5%, *p* < 0.001) and positive resection margins (28.0% vs. 9.4%, *p* = 0.019). The numbers of resected lymph nodes (14 (3–33) vs. 8 (2–36), *p* = 0.023) as well as the number of metastatic lymph nodes in the specimen (2 (0–15) vs. 0 (0–7), *p* < 0.001) were also higher in the disease-progression group. Of the *n* = 13 patients with positive resection margins, *n* = 7 (53.8%) showed disease progression. In this group, most common location of disease progression was the liver (*n* = 4), followed by recurrence in locoregional lymph nodes in *n* = 2 patients and pancreatic recurrence in *n* = 1 patient. Not surprisingly, only patients with progression received additional therapies (88.0% vs. 0.0%, *p* < 0.001) including SSA (*n* = 11, 44.0%) PRRT (*n* = 8, 32.0%), everolimus (*n* = 4, 16.0%), chemotherapy (*n* = 12, 48.0%) and brachytherapy (*n* = 5, 20.0%).

**Table 1:** Comparison between pNET patients with and without postoperative progression

		Total (*n* = 89)	No progression (*n* = 64)	Progression (*n* = 25)	*p*-value
Sex^1^	Male	44 (49.4)	32 (50.0)	12 (48.0)	0.865
	Female	45 (50.6)	32 (50.0)	13 (52.0)	
Age [years]^2^		62 (21–82)	64 (21–82)	54 (35–78)	0.079
BMI [kg/m^2^]^2^ (*n* = 80)		26.6 (15.6–40.5)	26.8 (15.6–40.5)	25.5 (18.7–38.6)	0.635
ASA^1^	1	4 (4.5)	3 (4.7)	1 (4.0)	0.125
	2	49 (55.1)	31 (48.4)	18 (72.0)	
	3	36 (40.4)	30 (46.9)	6 (24.0)	
Symptomatic^1^	Incidental	67 (75.3)	52 (81.3)	14 (61.0)	**0.025**
	Symptomatic	22 (24.7)	12 (18.8)	10 (39.0)	
UICC^1^	I	30 (33.7)	28 (43.8)	2 (8.0)	**< 0.001**
	II	35 (39.3)	28 (43.8)	7 (28.0)	
	III	24 (27.0)	8 (12.5)	16 (64.0)	
Surgical technique^1^	Open	63 (70.8)	40 (62.5)	23 (92.0)	**0.008**
	MIS	26 (29.2)	24 (37.5)	2 (8.0)	
Surgical procedure^1^	DP	57 (64.0)	48 (75.0)	9 (36.0)	**0.001**
	PPPD	31 (34.8)	16 (25.0)	15 (60.0)	
	Pancreatectomy	1 (1.1)	–	1 (4.0)	
Tumor location^1^	proximal	31 (34.8)	16 (25.0)	15 (60.0)	**0.002**
	distal	58 (65.2)	48 (75.0)	10 (40.0)	
Multifocality^1^		3 (3.4)	3 (4.7)	0 (0.0)	0.556
Grading (WHO)^1^	G1	44 (49.4)	40 (62.5)	4 (16.0)	**< 0.001**
	G2	38 (42.7)	23 (35.9)	15 (60.0)	
	G3	7 (7.9)	1 (1.6)	6 (24.0)	
Alternative grading^1^	G1	44 (49.4)	40 (62.5)	4 (16.0)	**< 0.001**
	G2a	27 (30.3)	19 (29.7)	8 (32.0)	
	G2b	11 (12.4)	4 (6.3)	7 (28.0)	
	G3	7 (7.9)	1 (1.6)	6 (24.0)	
Ki-67 [%]^2^		3 (1–68)	2 (1–50)	10 (1–68)	**< 0.001**
T stage^1^	T1	32 (36.0)	28 (43.8)	4 (16.0)	**0.002**
	T2	28 (31.5)	22 (34.4)	6 (24.0)	
	T3	29 (32.6)	14 (21.9)	15 (60.0)	
	T4	0 (0.0)	0 (0.0)	0 (0.0)	
Tumor size [mm] ^2^		25 (2–140)	18 (2–120)	37 (15–140)	**< 0.001**
N stage^1^	N0	64 (71.9)	56 (87.5)	8 (32.0)	**< 0.001**
	N+	25 (28.1)	8 (12.5)	17 (69.0)	
Lymph nodes resected^2^		10 (2–36)	8 (2–36)	14 (3–33)	**0.023**
Lymph nodes positive^2^		0 (0–15)	0 (0–7)	2 (0–15)	**< 0.001**
R status^1^	R0	75 (84.3)	56 (90.6)	17 (68.0)	**0.019**
	R+	13 (14.6)	6 (9.4)	7 (28.0)	
	Rx	1 (1.1)	0 (0.0)	1 (4.0)	
Additional treatment		22 (24.7)	0 (0.0)	22 (88.0)	**< 0.001**
	Chemotherapy	12 (13.5)	0 (0.0)	12 (48.0)	
	SSA	11 (12.3)	0 (0.0)	11 (44.0)	
	PRRT	8 (9.0)	0 (0.0)	8 (32.0)	
	Everolimus	4 (4.5)	0 (0.0)	4 (16.0)	
	Brachytherapy	5 (5.6)	0 (0.0)	5 (20.0)	
	SIRT	3 (3.3)	0 (0.0)	3 (12.0)	
	TAE	1 (1.1)	0 (0.0)	1 (4.0)	

^1^Data shown as frequencies (percentage); ^2^median (range); pNET, pancreatic neuroendocrine tumor; BMI, Body Mass Index; ASA, Association of Anesthesiologists; UICC, Union for International Cancer Control; MIS, minimally invasive surgery; DP, distal pancreatectomy; PPPD, pylorus preserving pancreaticoduodenectomy; SSA, Somatostatin analogue; PRRT, Peptide receptor radionuclide therapy; TAE, Transarterial embolization; SIRT, Selective internal radiation therapy.

Prognostic value of WHO and alternative tumor grading.

**Fig. 1:**
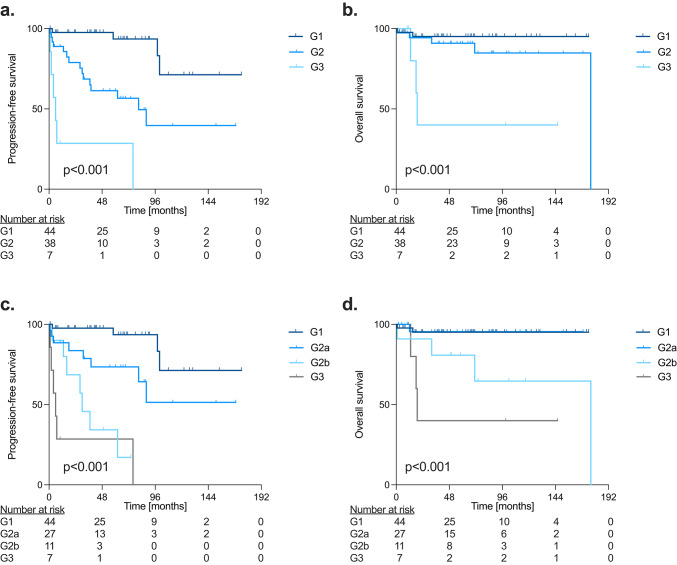
Comparison of progression-free survival (PFS) and overall survival (OS) in pNET patients. **a** PFS according to grading differs in whole-group (*p* < 0.001) and pairwise comparisons (G1 vs. G2: *p* < 0.001, G2 vs. G3: *p* < 0.001); **b** OS according to grading differs in whole-group (*p* < 0.001) and between G2 and G3 tumors (*p* = 0.011); **c** PFS according to alternative grading differs in whole-group (*p* < 0.001) and pairwise comparisons (G1 vs. G2a: *p* = 0.014, G2a vs. G2b: *p* = 0.014); **d** OS according to alternative grading differs in whole-group (*p* < 0.001). Pairwise comparison indicates longer OS in G2a than in G2b tumors (*p* = 0.060). Survival rates were compared using log-rank tests. Censored data are marked with ticks

Survival analyses were performed to identify associations of the conventional WHO grading and the alternative grading with PFS and OS, as shown in Fig. 1a. to d. PFS differed significantly in the overall comparison of the 3 different grading groups with 5-year PFS rates of 93.6% in G1 patients, 61.4% in G2 patients and 28.6% in G3 patients (*p* < 0.001) (s. Figure 1a). The respective median estimated PFS was not reached during follow-up in the G1 group while it was 81 months (95%-CI 49.4-112.6) in G2 and 6 months (95%-CI 0.9–11.1) in G3. Additional pairwise comparisons showed significant differences regarding PFS between G1 and G2 (*p* < 0.001) and G2 and G3 (*p* < 0.001). Comparing OS in G1 and G2 showed no differences with 5-year OS rates of 95.2% in G1 and 91.0% in G2 (*p* = 0.296), while OS in G2 tumors was significantly longer than in G3 tumors (5-year OS rate: 40.0%) (*p* = 0.011).

Comparison of PFS in the grading groups of the alternative grading classification showed significant differences with 5-year PFS rates of 93.6% in G1 patients, 73.5% in G2a, 34.3% in G2b and 28.6% in G3 (*p* < 0.001). The respective median PFS was not reached during follow-up in G1 and G2a patients while it was 30 months (95%-CI 17.1–42.8) in G2b and 6 months (95%-CI 0.9–11.1) in G3 patients. Pairwise comparisons confirmed significant differences between G1 and G2a (*p* = 0.014) and G2a and G2b (*p* = 0.014). No difference in PFS was found between G2b and G3 (*p* = 0.192). The OS according to the alternative classification showed significant differences (*p* < 0.001). In pairwise comparisons, no significant differences were stated between G1 and G2a (5-year OS rates 95.2% vs. 95.5%, *p* = 0.923) and G2b and G3 tumors (5-year OS rates 80.8% vs. 40.0%, *p* = 0.255). However, G2a tumors showed a trend towards longer OS than G2b tumors (5-year OS rates 95.5% vs. 80.8%, *p* = 0.060).

Tumor location and disease progression in pNETs.

Since patients who presented with postoperative disease recurrence or progression more frequently presented with a proximal pancreatic tumor than patients without progression, a comparison depending on tumor location was conducted. As shown in Supplementary Table 2, basic demographic data such as sex, age, BMI or ASA status did not differ according to tumor location. Regarding tumor stage and histopathological features, patients with proximal tumors presented more frequently with stage III (48.4% vs. 15.5%) tumors (*p* = 0.003), had a higher proportion of lymph node metastases (48.4% vs. 17.2%, *p* = 0.002) and the number of resected lymph nodes was higher, too (12 (4–33) vs. 8 (2–36), *p* = 0.022). No statistically significant differences were stated in regards of grading, tumor size and T stage. As shown in Supplementary Fig. 1a, survival analysis identified shorter PFS in proximal (5-year PFS rate: 56.2%) than in distal pNETs (5-year PFS rate 85.0%), *p* = 0.003. No significant differences were found in OS according to tumor location with 5-year OS rates of 94.5% in distal and 80.8% in proximal tumors (*p* = 0.210) (s. Supplementary Fig. 1b).

However, after matching proximal and distal pNETs according to UICC tumor stage and age, no significant differences were stated regarding pathological data including grading, T stage, tumor size, N stage and R status (s. Supplementary Table 2). In addition, survival analyses of the matched cohorts showed similar PFS in proximal and distal pNETs with 5-year PFS rates of 61.6% vs. 78.5% (*p* = 0.460) and similar OS with 5-year OS rates of 76.0% vs. 95.5% (*p* = 0.199) (s. Figure Supplementary Fig. 2).

Alternative grading and nodal involvement predict progression in pNETs.

To identify independent factors associated with disease progression, univariate and multivariable Cox regression analysis were performed. In the univariate model proximal tumor location, (alternative) tumor grading, tumor size above 4 cm and lymph node metastases were identified as risk factors for impaired PFS (s. Table 2). As only 3 variables could be included in the multivariable analysis, the factors with the highest hazard ratios and lowest p values (≤ 0.001) were identified. After multivariable Cox regression analysis, alternative tumor grading (G2b: 8.396 (2.025–34.801), *p* = 0.003; G3: 17.649 (3.912–79.627), *p* < 0.001) and lymph node metastases (2.657 (1.040–6.789), *p* = 0.041) were confirmed as independent risk factors for shorter PFS.

**Table 2:** Univariate and multivariable Cox regression analysis for variables affecting PFS in stage I-III pNET patients

	Univariate		Multivariable	
	HR (95%-CI)	*p* value	HR (95%-CI)	*p* value
Female gender	1.227 (0.559–2.697)	0.610		
Age > 60 years	0.544 (0.244–1.211)	0.136		
ASA 3	0.542 (0.197–1.492)	0.236		
Symptomatic	1.879 (0.824–4.283)	0.134		
Proximal tumor	3.137 (1.408–6.992)	**0.005**		
*Alternative grading*				
G1	Reference		Reference	
G2a	4.114 (1.235–13.703)	**0.021**	2.917 (0.839–10.136)	0.092
G2b	17.048 (4.544–63.956)	**< 0.001**	8.396 (2.025–34.801)	**0.003**
G3	36.106 (9.205-141.629)	**< 0.001**	17.649 (3.912–79.627)	**< 0.001**
*T Status*				
T1	Reference			
T2	1.421 (0.397–5.090)	0.589		
T3	7.417 (2.415–22.777)	**< 0.001**		
Tumor diameter > 4 cm	4.256 (1.882–9.621)	**< 0.001**	2.013 (0.803–5.045)	0.135
Positive N status	6.330 (2.724–14.707)	**< 0.001**	2.657 (1.040–6.789)	**0.041**
Positive R Status	3.633 (1.494–8.838)	**0.004**		

pNET, pancreatic neuroendocrine tumor; ASA, Association of Anesthesiologists.; MIS, minimally invasive surgery.

## Discussion

Identification of risk factors for disease recurrence and progression in patients undergoing surgery for pNETs is crucial for choosing the adequate and personalized therapy and follow-up strategy. In the current study, tumor stages, grading and tumor related symptoms were associated with postoperative progression after pancreatic resection in a large single-center pNET cohort including stage I to III patients. Survival analyses demonstrated that both, the conventional WHO and the alternative grading classifications were strongly associated with PFS, with higher grades correlating with shorter PFS. Cox regression analyses revealed lymph node metastases and tumor grading (particularly G2b and G3 according to the alternative grading classification) as independent predictors of disease progression.

Tumor biology and pathological features have been previously associated with outcome prediction and disease recurrence or progression in pNETs [10–12]. WHO tumor grade based on the Ki-67 index is a well-established predictor of recurrence or progression and survival in pNETs [17, 18]. However, discussion of the defined Ki-67 cut-off values in pNET, but also in NET with other origins, is ongoing [10, 14, 19, 20]. In the current study, we analyzed an alternative grading classification based on Ki-67 recently proposed by Eren et al. for pNET [9] and Reinhard et al. in small intestinal NET [20]. It subdivides the G2 group into G2a with Ki-67 index ranging from 3% to below 10% and G2b with Ki-67 from 10% to 20%. We were able to show that G2b proportions were higher in patients with postoperative progression and disease trajectory differed between G2a and G2b tumors. PFS and OS in patients with G2b tumors were similar to those with G3 tumors and OS was similar in G1 and G2a patients. These findings suggest that G2 tumors do not behave uniformly. They seem to consist of both indolent (G2a) and more aggressive (G2b) subtypes that resemble G3 tumors. Cox regression analysis identified this alternative grading classification along with lymph node metastases as independent risk factors for disease recurrence and progression. The hazard ratio for impaired PFS increased with each higher grading category from 2.917 in G2a tumors, to 8.396 in G2b up to 17.649 in G3 tumors. These results are in line with the findings from Eren et al. who identified more aggressive tumor features in the G2b group with regards to metastatic behavior including lymph node and distant metastases as well as histopathological features such as tumor size and perineural and vascular invasion [9]. Analogous to the findings presented here, they concluded that the G2b group therefore more closely resembled G3 tumors. Subdivision of G2 pNETs into G2a with a less aggressive and G2b with a more aggressive phenotype and higher risk for disease recurrence should impact the choice of follow-up management and a possible evaluation of (neo-)adjuvant treatment in surgical pNET patients.

Evidence on adjuvant treatment in pNETs is scarce and conflicting. In a retrospective analysis, Barrett et al. evaluated the effect of adjuvant therapy with either chemotherapy or SSA on survival compared to surveillance [21]. They found no positive effect of adjuvant therapy on neither recurrence-free survival (RFS) nor OS and identified shorter RFS in patients who received either chemotherapy or SSA compared to those who did not. However, it is most likely that patients with more aggressive tumors received chemotherapy more often, while those with less aggressive tumors underwent surveillance. Currently, a randomized phase II trial is comparing the efficacy of capecitabine and temozolomide versus observation in high-risk pNET patients but the results are still pending [22]. Guidelines acknowledge this lack of evidence, too: current European and American guidelines do not recommend adjuvant treatment in resected pNETs [15, 23] although ENETS recommendations consider adjuvant treatment for “high risk” patients, preferably in clinical trials. In addition to G3 tumors, the presented data suggest that G2b tumors should also be considered high-risk. Distinguishing the G2 group into G2a and G2b allows for more personalized treatment decisions within the existing vague guideline recommendations.

Furthermore, considering the shorter PFS, shorter inter-imaging intervals during follow-up for G2b tumors (alongside with G3 tumors) might be appropriate and should be considered.

Moreover, the role of neoadjuvant treatment in pNET is unclear. The NEOLUPANET trial investigated the use of neoadjuvant PRRT with 177Lu-DOTATATE before surgery in patients with pNETs at high risk for recurrence. This is defined as having at least one feature such as tumor size greater than 4 cm, involvement of adjacent organs, vascular invasion, mesenteric, portal or splenic vein thrombosis, liver and lymph node metastases and a Ki-67 greater than 10% [24]. Although data on oncological outcomes and a control group are lacking, the study demonstrated the safety and effectiveness of preoperative PRRT leading to a partial response in 18 of 31 patients and stable disease in the remaining 13. Ki-67 index above 10% was recognized as a risk factor for disease recurrence and therefore, further subdividing the heterogeneous G2 group could help determine which patients are eligible for neoadjuvant treatment.

Lastly, distinguishing between G2a and G2b could be useful when evaluating treatment for small non-functional pNETs measuring between 1 and 2 cm. Recent data support the safety of surveillance when “aggressive features” are absent and ENETS guidelines recommend personalized management for this group [15, 25, 26]. Due to the higher risk of disease recurrence, surgery appears to be the appropriate treatment for patients with G2b tumors measuring between 1 and 2 cm.

In line with previous findings, UICC stage, tumor size and nodal involvement were associated with postoperative progression [27–30]. On the other hand, no association of sex or age with progression was identified. There has been ongoing discussion about age-adjusted management also in pNETs with partly conflicting results [12, 31]. The presented data do not favor adjustment of treatment aggressiveness or different follow-up strategies based on patients’ age. Therapy and follow-up strategies, however, should always consider patient-specific characteristics and overall performance besides the tumor-specific factors.

We identified patients with proximal pNETs at a higher risk for tumor progression and shorter PFS in our cohort compared to distal tumors before matching. They also showed higher proportions of stage III tumors and lymph node metastases. After propensity score matching, these differences were not stated anymore and neither PFS nor OS differed according to tumor location. A proximal location of the tumor has been previously described as risk factor for recurrent disease [28, 32] and shorter OS [18, 33]. In addition, similar to the here-presented data, proximal pNETs were shown to have increased risk for lymph node metastases than pNETs located in the body and tail [32, 34]. As the association between tumor location and clinical outcomes in pNETs remains inconclusive, further data and studies are needed to clarify its role and potential utility in risk stratification.

There are certain limitations to the nature of this study which should be acknowledged. First, this includes the retrospective design of the study, which inherently carries the risk of selection bias and incomplete data. Further, the size of the study population is limited due to the rarity of the disease. This may especially affect the statistical analysis of subgroups, given the small cohort sizes. As the analysis was restricted to surgical patients, the generalizability of the presented findings may be limited in non-surgical populations. Given that pNETs are rare tumors with diverse characteristics (such as functionality, multifocality, and associations with hereditary syndromes) it can be challenging to assemble a study cohort that is both sufficiently large and clinically comparable. This remains true even at specialized NET centers. To tackle these limitations, larger multi-center studies should be promoted.

## Conclusions

This study identified tumor grading and lymph node metastases as risk factors for disease progression in patients without distant metastases undergoing surgery for pNETs. In addition, subdivision of G2 tumors into G2a with Ki-67 from 3 to < 10% and G2b with Ki-67 from 10 to 20% may allow for more accurate individual risk stratification for disease progression and therefore should be included in individual treatment decisions.

## Supplementary Information

Below is the link to the electronic supplementary material.

Supplementary Material 1

## Data Availability

The datasets generated during and analyzed during the current study are not publicly available due to reasons of sensitivity and are only available from the corresponding author on reasonable request.
