# Supplementary material for: Predictors of disease progression in pancreatic neuroendocrine tumors after surgery
Source: Updates Surg. 2026 Feb 16;78(3):1253–61. doi: 10.1007/s13304-026-02521-0 (PMC13249762; doi:10.1007/s13304-026-02521-0)
Supplement: Supplementary file 1 — Supplementary Material 1 [file 13304_2026_2521_MOESM1_ESM.docx]

**Supplementary Material**

**Predictors of disease progression in pancreatic neuroendocrine tumors after surgery**

***Journal: Updates in Surgery***

Eva Maria Dobrindt^1*^, Janina Maren Krömer^1*^, Martina T. Mogl^1^, Agata Dukaczewska^1^, Charlotte Friederieke Müller-Debus^1^, Peter Steinhagen^2^, Uli Fehrenbach^3^, Thomas Malinka^1^, Johann Pratschke^1^, Frederike Butz^1°^

^1^Department of Surgery, Campus Charité Mitte | Campus Virchow Klinikum, Charité – Universitätsmedizin Berlin, Corporate Member of Freie Universität Berlin and Humboldt-Universität zu Berlin, Berlin, Germany

^2^Department of Hepatology and Gastroenterology, Campus Charité Mitte | Campus Virchow-Klinikum, Charité – Universitätsmedizin Berlin, Corporate Member of Freie Universität Berlin and Humboldt-Universität zu Berlin, Berlin, Germany

^3^Department of Radiology, Charité – Universitätsmedizin Berlin, Corporate Member of Freie Universität Berlin and Humboldt-Universität zu Berlin, Berlin, Germany

* These authors contributed equally

**° Corresponding author:**

Dr. med. Frederike Butz

Department of Surgery, Charité Campus Mitte

Charitéplatz 1, 10117 Berlin, Germany

frederike.butz@charite.de

Supplementary Table 1 Comparison of pNET patients depending on tumor location

|  |  | Proximal  (n = 31) | Distal  (n = 58) | p-value |  |
| --- | --- | --- | --- | --- | --- |
| Sex^1^ | Male | 15 (48.4) | 29 (50.0) | 0.885 |  |
|  | Female | 16 (45.9) | 29 (50.0) |  |  |
| Age [years]^2^ |  | 64 (35-76) | 61 (21-82) | 0.983 |  |
| BMI [kg/m^2^]^2^ (n = 80) | | 27.0 (15.6 - 38.6) | 25.6 (15.6-40.5) | 0.524 |  |
| ASA^1^ | 1 | 2 (6.5) | 2 (3.4) | 0.766 |  |
|  | 2 | 16 (51.6) | 33 (56.9) |  |  |
|  | 3 | 13 (41.9) | 23 (39.7) |  |  |
| UICC^1^ | I | 9 (29.0) | 21 (36.2) | **0.003** |  |
|  | II | 7 (22.6) | 28 (48.3) |  |  |
|  | III | 15 (48.4) | 9 (15.5) |  |  |
| Surgical procedure^1^ | DP | 0 (0.0) | 57 (98.3) | **<0.001** |  |
|  | PPPD | 31 (100.0) | 0 (0.0) |  |  |
|  | Pancreatectomy | 0 (0.0) | 1 (1.7) |  |  |
| Multifocality^1^ | | 1 (3.2) | 2 (3.4) | 1.0 |  |
| Grading (WHO)^1^ | G1 | 16 (51.6) | 28 (48.3) | 0.810 |  |
|  | G2 | 12 (38.7) | 26 (44.8) |  |  |
|  | G3 | 3 (9.7) | 4 (6.9) |  |  |
| Alternative grading^1^ | G1 | 16 (51.6) | 28 (48.3) | 0.908 | |
|  | G2a | 9 (29.0) | 18 (31.0) |  |  |
|  | G2b | 3 (9.7) | 8 (13.8) |  |  |
|  | G3 | 3 (9.7) | 4 (6.9) |  |  |
| Ki-67 [%]^2^ |  | 2 (1-68) | 3 (1-60) | 0.623 |  |
| T stage^1^ | T1 | 11 (35.5) | 21 (36.2) | 0.291 |  |
|  | T2 | 7 (22.6) | 21 (36.2) |  |  |
|  | T3 | 13 (41.9) | 16 (27.6) |  |  |
|  | T4 | 0 (0.0) | 0 (0.0) |  |  |
| Tumor size [mm]^2^ | | 25 (4-140) | 25 (2-120) | 0.667 |  |
| N stage^1^ | N0 | 16 (51.6) | 48 (82.8) | **0.002** |  |
|  | N+ | 15 (48.4) | 10 (17.2) |  |  |
| Lymph nodes resected^2^ | | 12 (4-33) | 8 (2-36) | **0.022** |  |
| Lymph nodes positive^2^ | | 1 (0-15) | 0 (0-10) | **0.003** |  |
| R status^1^ (n = 88) | R0 | 23 (74.2) | 52 (89.7) | 0.075 |  |
|  | R+ | 8 (25.8) | 5 (8.6) |  |  |
|  | Rx | 0 0.0) | 1 (1.7) |  |  |
| Additional treatment ^1^ | | 13 (41.9) | 9 (15.5) | **0.009** |  |
| Progress^1^ | | 15 (48.4) | 10 (17.2) | **0.003** |  |
| NET-related death^1^ | | 5 (16.1) | 5 (8.6) | 0.308 |  |

^1^Data shown as frequencies (percentage); ^2^median (range); pNET, pancreatic neuroendocrine tumor; BMI, Body Mass Index; ASA, Association of Anesthesiologists; UICC, Union for International Cancer Control; MIS, minimally invasive surgery; SSA, Somatostatin analogue; PRRT, Peptide receptor radionuclide therapy; TAE, Transarterial embolization; SIRT, Selective internal radiation therapy.

**a.**  **b.**

Supplementary Figure 1 Comparison of **a.** Progression-free survival (PFS) and **b.** Overall survival (OS) in pNET patients depending on tumor location. Patients with proximal pNETs showed shorter PFS (p=0.003) while OS did not differ significantly according to tumor location (p=0.210). Survival rates were compared using log-rank tests. Censored data are marked with ticks.

Supplementary Table 2 Comparison of pNET patients depending on tumor location – matched study cohort

|  |  | Proximal  (n = 25) | Distal  (n = 25) | p-value |
| --- | --- | --- | --- | --- |
| Sex^1^ | Male | 11 (44.0) | 12 (48.0) | 0.777 |
|  | Female | 14 (56.0) | 13 (52.0) |  |
| Age [years]^2^ |  | 65 (35 – 74) | 65 (31 – 78) | 0.877 |
| BMI [kg/m^2^]^2^ | | 26.9 (15.6-33.4) | 27.7 (15.6-39.9) | 0.481 |
| ASA^1^ | 1 | 1 (4.0) | 0 (0.0) | 1.0 |
|  | 2 | 13 (52.0) | 14 (56.0) |  |
|  | 3 | 11 (44.0) | 11 (44.0) |  |
| UICC^1^ | I | 9 (36.0) | 8 (32.0) | 0.939 |
|  | II | 7 (28.0) | 8 (32.0) |  |
|  | III | 9 (36.0) | 9 (36.0) |  |
| Multifocality^1^ | | 1 (4.0) | 0 (0.0) | 1.0 |
| Grading (WHO)^1^ | G1 | 15 (60.0) | 9 (36.0) | 0.252 |
|  | G2 | 8 (32.0) | 13 (42.0) |  |
|  | G3 | 2 (8.0) | 3 (12.0) |  |
| Alternative grading^1^ | G1 | 15 (60.0) | 9 (36.0) | 0.458 |
|  | G2a | 5 (20.0) | 8 (32.0) |  |
|  | G2b | 3 (12.0) | 5 (20.0) |  |
|  | G3 | 2 (8.0) | 3 (12.0) |  |
| Ki-67 [%]^2^ |  | 2 (1-68) | 4 (1-60) | 0.184 |
| T stage^1^ | T1 | 9 (36.0) | 8 (32.0) | 1.0 |
|  | T2 | 6 (24.0) | 6 (24.0) |  |
|  | T3 | 10 (40.0) | 11 (44.0) |  |
|  | T4 | - | - |  |
| Tumor size [mm]^2^ | | 23 (2-60) | 32.5 (5-140) | 0.054 |
| N stage^1^ | N0 | 16 (64.0) | 16 (64.0) | 1.0 |
|  | N+ | 9 (36.0) | 9 (36.0) |  |
| Lymph nodes resected^2^ | | 11 (4-33) | 8 (2-20) | 0.221 |
| Lymph nodes positive^2^ | | 0 (0-15) | 0 (0-10) | 0.747 |
| R status^1^ | R0 | 20 (80.0) | 23 (92.0) | 0.417 |
|  | R+ | 5 (20.0) | 2 (8.0) |  |
| Adjuvant Therapy^1^ | | 8 (32.0) | 7 (28.0% | 0.758 |
| Progress^1^ | | 9 (36.0) | 7 (28.0) | 0.544 |
| NET-related death^1^ | | 5 (20.0) | 2 (8.0) | 0.417 |

^1^Data shown as frequencies (percentage); ^2^median (range); pNET, pancreatic neuroendocrine tumor; BMI, Body Mass Index; ASA, Association of Anesthesiologists; UICC, Union for International Cancer Control; MIS, minimally invasive surgery; SSA, Somatostatin analogue; PRRT, Peptide receptor radionuclide therapy; TAE, Transarterial embolization; SIRT, Selective internal radiation therapy.

**a.**  **b.**

Supplementary Figure 2 Comparison of **a.** progression-free survival (PFS) and **b.** overall survival (OS) in matched pNET patients depending on tumor location. PFS (p=0.460) and OS (p=0.199) did not differ significantly according to tumor location. Survival rates were compared using log-rank tests. Censored data are marked with ticks.
